# Updates on HTLV-1 Uveitis

**DOI:** 10.3390/v14040794

**Published:** 2022-04-12

**Authors:** Koju Kamoi, Toshiki Watanabe, Kaoru Uchimaru, Akihiko Okayama, Seiko Kato, Toyotaka Kawamata, Hisako Kurozumi-Karube, Noe Horiguchi, Yuan Zong, Yoshihisa Yamano, Isao Hamaguchi, Yasuhito Nannya, Arinobu Tojo, Kyoko Ohno-Matsui

**Affiliations:** 1 Department of Ophthalmology & Visual Science, Graduate School of Medical and Dental Sciences, Tokyo Medical and Dental University, Tokyo 113-8510, Japan; hkalbe@hotmail.co.jp (H.K.-K.); noegucchi@hotmail.co.jp (N.H.); zongyuan666@gmail.com (Y.Z.); k.ohno.oph@tmd.ac.jp (K.O.-M.); 2 Department of Hematology/Oncology, Research Hospital, The Institute of Medical Science, The University of Tokyo, Tokyo 108-8639, Japan; tnabe@marianna-u.ac.jp (T.W.); uchimaru@cbms.k.u-tokyo.ac.jp (K.U.); k-seiko@ims.u-tokyo.ac.jp (S.K.); toyotaka@ims.u-tokyo.ac.jp (T.K.); yasuhito.nannya@gmail.com (Y.N.); tojo.adm@tmd.ac.jp (A.T.); 3 Department of Practical Management of Medical Information, St. Marianna University School of Medicine, Kanagawa 216-8512, Japan; 4 Department of Medical Computational Biology and Genome Sciences, Laboratory of Tumor Cell Biology, Graduate School of Frontier Sciences, The University of Tokyo, Tokyo 108-8639, Japan; 5 Department of Rheumatology, Infectious Diseases and Laboratory Medicine, Faculty of Medicine, University of Miyazaki, Miyazaki 889-1601, Japan; okayama@miyakenkou.or.jp; 6 Division of Neurology, Department of Internal Medicine, St. Marianna University School of Medicine, Kanagawa 216-8511, Japan; yyamano@marianna-u.ac.jp; 7 Department of Safety Research on Blood and Biological Products, National Institute of Infectious Diseases, Tokyo 208-0011, Japan; 130hama@nih.go.jp; 8 Institute of Innovation Advancement, Tokyo Medical and Dental University, Tokyo 113-8510, Japan

**Keywords:** Human T-cell lymphotropic virus, HTLV-1 uveitis, horizontal transmission, Graves’ disease, biologics

## Abstract

HTLV-1 uveitis (HU) is the third clinical entity to be designated as an HTLV-1-associated disease. Although HU is considered to be the second-most frequent HTLV-1-associated disease in Japan, information on HU is limited compared to that on adult T-cell leukemia/lymphoma (ATL) and HTLV-1-associated myelopathy (HAM). Recent studies have addressed several long-standing uncertainties about HU. HTLV-1-related diseases are known to be caused mainly through vertical transmission (mother-to-child transmission), but emerging HTLV-1 infection by horizontal transmission (such as sexual transmission) has become a major problem in metropolitan areas, such as Tokyo, Japan. Investigation in Tokyo showed that horizontal transmission of HTLV-1 was responsible for HU with severe and persistent ocular inflammation. The development of ATL and HAM is known to be related to a high provirus load and hence involves a long latency period. On the other hand, factors contributing to the development of HU are poorly understood. Recent investigations revealed that severe HU occurs against a background of Graves’ disease despite a low provirus load and short latency period. This review highlights the recent knowledge on HU and provides an update on the topic of HU in consideration of a recent nationwide survey.

## 1. Introduction

Human T-lymphotropic virus type 1 (HTLV-1) was the first retrovirus identified to cause human diseases [1,2]. The focus on this virus had been increasing, and the World Health Organization recently published a technical report on HTLV-1 [3]. HTLV-1 is thought to infect at least 5–10 million people worldwide and approximately 1 million people in Japan [4].

Since the discovery of HTLV-1, several clinical entities of HTLV-1-associated disease have been identified, such as adult T-cell leukemia/lymphoma (ATL) [5,6,7], HTLV-1-associated myelopathy (HAM) [8,9], and HTLV-1 uveitis (HU) [10,11,12,13]. ATL is the most frequent HTLV-1-associated disease and is one of the most refractory types of leukemia/lymphoma [14]. In terms of the other HTLV-1-associated diseases, although epidemiological studies on HU are scant, cross-sectional studies in Japan have revealed a prevalence of HU of 112.2 per 100,000 HTLV-1 carriers [15], whereas the prevalence of HAM was reported to be 68.3 per 100,000 HTLV-1 carriers [16]. These cross-sectional surveys suggest that HU is the second-most frequent HTLV-1-associated disease following ATL, at least in Japan, because the prevalence of HU in other HTLV-1 endemic regions is unknown.

The characteristics of HU have been determined by various clinical and basic research studies three decades ago [17,18,19], and this clinical entity has since been regularly treated by ophthalmologists [20]. In fact, HU has been reported all over the world, including its recent discovery among Australian Aboriginal adults [21,22,23,24,25]. In the southern parts of Japan, HU is the most frequent type of uveitis and is considered one of the most important ocular diseases in endemic areas [26,27].

Although three decades have passed since the initial awareness of HU, several uncertainties related to this condition remain. Recently, several notable findings addressing these uncertainties were reported. This review highlights the latest information on HU and provides an update on the topic of HU in consideration of a recent nationwide survey in Japan.

## 2. Clinical Features of HU

HU is defined as intraocular inflammation caused by HTLV-1 infection [19], in which HTLV-1-infected T-cells disrupt the blood–ocular barrier, resulting in inflammation in the eye [28]. The main symptoms in patients at initial presentation include foggy vision, ocular floaters, blurring of vision, ocular hyperemia, ocular pain, and photophobia [26]. The main clinicopathological features of HU are vitreous opacity and retinal vasculitis [17,29,30].

Vitreous opacity is the most frequent ocular feature and is responsible for foggy vision, floaters, blurring of vision, and vision loss. Retinal vasculitis, identified as vascular leakage on fluorescence angiography, is also seen frequently, and it affects retinal function, resulting in impaired vision. Infiltrating cells, including HTLV-1-infected cells and inflammatory cells, which damage intraocular tissues, are seen in the anterior chamber and vitreous. Recurrence of HU is seen in around half of HU patients, leading to irreversible vision loss [26,31,32].

The current therapy for HU includes steroids through topical, sub-Tenon, and systemic administration. For focal treatment, topical steroid eye drops are used initially, followed by sub-Tenon injection of the steroid in unresponsive cases. However, systemic treatment (oral steroids and/or steroid pulse therapy) is needed in more than 30% of HU patients [19].

The reported ocular complications of HU include glaucoma, cataract, cystoid macular edema, and keratoconjunctivitis sicca [26]. In terms of systemic complications, an association between HU and hyperthyroidism due to Graves’s disease has been reported [26,33,34,35,36]. Additionally, in terms of concurrence of other HTLV-1-associated diseases, HAM is sometimes seen in HU patients [10,26,36], although ATL rarely occurs along with HU [26,36].

## 3. Tackling HTLV-1 in Ophthalmology: Nationwide Survey in Japan

Japan has approximately 1 million HTLV-1-infected individuals and is considered the most HTLV-1 endemic country among developed nations [37]. In the late 1980s and early 1990s, clinical and laboratory data from Japan identified HTLV-1 as a cause of uveitis, which led to the identification of the third clinical entity among HTLV-1-associated diseases, i.e., HTLV-1 uveitis (HU) [19]. Since then, ophthalmologists in Japan have been actively studying HU and have been providing new insights into HU through conferences and publications.

Our latest nationwide survey of ophthalmic care for HTLV-1 in Japan provided new information and identified several concerns related to HU [20]. The survey found that 58.3% of regional core facilities in Japan routinely measure HTLV-1 antibodies when diagnosing uveitis (Table 1), with the number increasing to 86.8% of facilities when counting facilities that perform such measurements when an HTLV-1-related ocular manifestation is suspected (Table 2).

This high percentage of testing for HTLV-1 antibodies in ophthalmology is unique to Japan. In the three decades since the establishment of the entity of HU, the majority of facilities in Japan started performing tests for HTLV-1 antibodies when evaluating the differential diagnoses of uveitis. This implies that ophthalmologists in Japan are well aware of HTLV-1 infection (Table 1 and Table 2).

In Japan, HTLV-1 antibody screening of all donated blood has been conducted by the Japanese Red Cross since the latter half of the 1980s. Since then, the implementation of infection control measures has contributed to eliminating HTLV-1 infections. Acknowledging the importance of HTLV-1 infection, the Japanese government also started routine antenatal HTLV-1 antibody screening of pregnant women in 2011 as one of the priority measures of “Anti-HTLV-1 Initiatives by the Japanese Government” [38,39]. These measures against HTLV-1 infection have mitigated horizontal transmissions (infusion-related infection), and most vertical transmissions (breastfeeding-related infection) of HTLV-1. However, the prevalence of HTLV-1 infection in Japan has not decreased significantly, and there are still approximately 1 million HTLV-1 carriers in Japan. In concurrence with this observation, our nationwide survey also found no decline in the number of new patients with HU in 87.3% of facilities [20].

## 4. Transmission Route for the Development of HU

The lack of a measurable decrease in the number of new patients with HU in our nationwide survey could be explained by referring to a recent study by the Japanese Red Cross Blood Center [20,40]. A Japanese Red Cross data-based study focused on horizontal transmission found that the number of new HTLV-1-infected individuals was approximately 4000 persons/year in Japan and was highest among women aged 50–59 years and men aged 60–69 years in the Tokyo metropolitan region [40]. This suggests that currently, the noteworthy transmission route is horizontal transmission, which probably accounts for the recent non-decreasing trend in new HU patients.

This is contrary to the transmission route for the other HTLV-1-associated diseases, ATL and infective dermatitis, which are believed to mainly occur through a vertical transmission route [41,42]. So far, because there was no information on the transmission route of HU, the long-held belief was that HU occurred through vertical transmission, similar to ATL and infective dermatitis.

Considering the lack of a decline in the incidence of HU in our nationwide survey, despite attempts to eliminate the transmission route described above by the Japanese Red Cross and government, we suspected that there must be a horizontal transmission route for the development of HU and started investigating the transmission route for HU patients and investigating their long-term clinical course.

**Figure 1:**
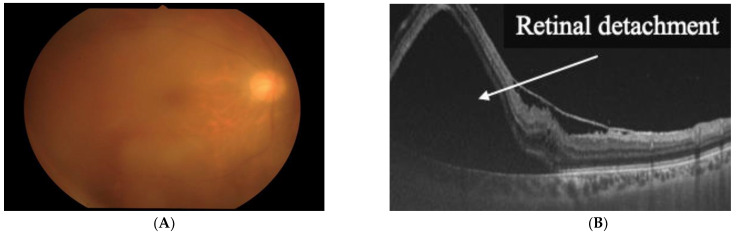
Horizontal transmission of HTLV-1 is responsible for HTLV-1 uveitis. (**A**) Vitreous opacity is seen in the right eye. (**B**) Severe and persistent ocular inflammation of horizontally transmitted HU resulted in retinal detachment (Reproduced with permission from Kamoi et al., *Lancet Infect. Dis*. 2021 [44]).

By analyzing the HTLV-1 infectious status of HU patients’ families and the clinical manifestations of HU patients during medical care, we determined that a horizontal transmission route was responsible for HU. In addition, we observed that horizontally transmitted HU caused severe and persistent ocular inflammation during follow-up (Figure 1) [43].

In the current environment of the non-declining trend of HTLV-1 infections, the existence of horizontal transmission of HU should be acknowledged because it can result in more severe inflammation than vertical transmission. Thus, physicians should take the route of infection into consideration when providing medical care to patients with HU.

## 5. Factors Associated with the Development of HU

Clinically, the proviral load (the percentage of infected peripheral blood mononuclear cells) is one of the most important factors in the development of HTLV-1-associated diseases [45]. In terms of the time course, the proviral load is low at the initial stages of infection but gradually increases during the chronic stage of infection [45]. A high provirus load is closely related to the development of ATL and HAM [45,46,47]. However, little is known about the time course of the development of HU and the factors contributing to it.

**Figure 2:**
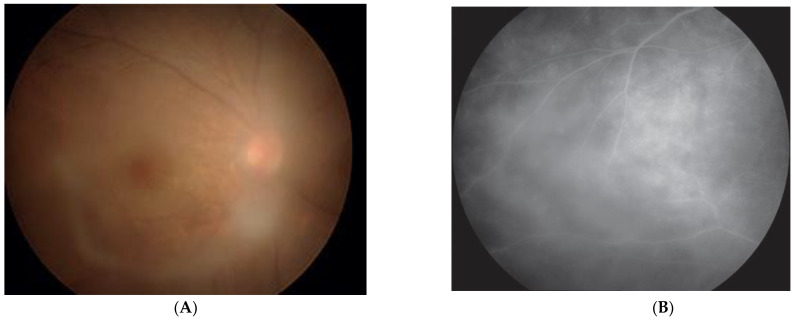
HU occurred after methimazole treatment following the onset of Graves’ disease. (**A**) Vitreous opacity is seen in the right eye. (**B**) Retinal vasculitis can be seen by fluorescein angiography (Reproduced with permission from Kamoi et al., *Lancet* 2022 [43]).

Through virologic analysis of HU patients, along with systemic investigations, we found that severe HU occurred in young HTLV-1 carriers after the onset of Graves’ disease and subsequent administration of methimazole, even though the provirus load was low [19] (Figure 2).

This phenomenon implies that the acceleration of the development of HU occurs in HTLV-1-infected patients who develop Graves’ disease and receive methimazole treatment despite a low provirus load and short latency period. This observation suggests the need for physicians to be cautious when Graves’ disease patients complain of blurry vision and that physicians, including endocrinologists and ophthalmologists, should rule out HTLV-1 infection in the differential diagnoses in such patients.

A possible mechanism for this association between HU and Grave’s disease is that thyroid hormones and methimazole might stimulate a small number of circulating HTLV-1-infected cells, which might contribute to the breakdown of the blood–ocular barrier, resulting in severe ocular inflammation. However, further analysis is needed to clarify this phenomenon.

## 6. Safety of Biologics in Terms of the Eye in HTLV-1 Infection

Biologics, such as tumor necrosis factor (TNF)-α antibody, were introduced in the late 1990s as a molecularly targeted therapy for inflammation [48,49,50,51,52]. Biologics have been proven to be effective in suppressing inflammation and are currently used for a wide range of inflammatory diseases [53]. However, a paradoxical response or exacerbation of inflammation is also well known during anti-TNF-α therapy [54]. In particular, the occurrence of uveitis (ocular inflammation) has been reported as a major paradoxical effect [55].

It is estimated that there is a large number of patients with inflammatory diseases, such as rheumatoid arthritis, who need to receive biologics despite having HTLV-1 infection [56]. However, patients with rheumatoid arthritis and HTLV-1 infection are susceptible to developing exacerbation of HTLV-1-associated conditions, such as HTLV-1-associated myopathy and HU, after the use of biological agents, such as tocilizumab, as was previously described in a case report [57]. However, none of the existing rheumatology, gastroenterology, or ophthalmology-related international guidelines recommend assessments for HTLV-1 infection before the administration of biologics.

In the field of ophthalmology, no evidence has been found for prescribing biologics to HTLV-1 carriers. In fact, our nationwide survey determined that the measurement of HTLV-1 antibodies is not performed in 50.0% of facilities prescribing biologics, and the reason commonly stated for this was a lack of knowledge regarding whether or not the measurement should be performed [20].

In response to this survey, we started investigating the effects of biologics on the eye under HTLV-1 infectious conditions in vitro. At first, we focused on infliximab, the first biologic for inflammatory diseases [58], and found that infliximab does not exacerbate HTLV-1-associated inflammatory conditions in the eye [59]. Next, we assessed adalimumab, the most frequently used biologic [53], and observed that adalimumab also does not exacerbate HTLV-1-associated inflammation in the eye [60].

Although these reports provided new evidence regarding the ocular safety of the use of biologics for inflammatory diseases in HTLV-1 carriers, which would be clinically useful for ophthalmologists, further analysis, including evaluation of other biologics, is needed in the future.

## 7. Ocular Manifestations of HTLV-1-Associated Diseases

Although HU is the most well-known and most studied ocular manifestation of HTLV-1, the other HTLV-1-associated disease entities might also present ocular symptoms. Several surveys that enrolled a large number of patients found that HAM sometimes occurs along with HU [10,26,36]. However, information on ATL-related ocular manifestations is scant, and only sporadic cases have been reported because of their rarity. Therefore, we performed a nationwide survey of the ocular manifestations of ATL and found that the most frequent manifestation is intraocular infiltration, followed by opportunistic infection (with all cases involving cytomegalovirus (CMV) retinitis), keratoconjunctivitis sicca, and scleritis [61] (Table 3).

**Figure 3:**
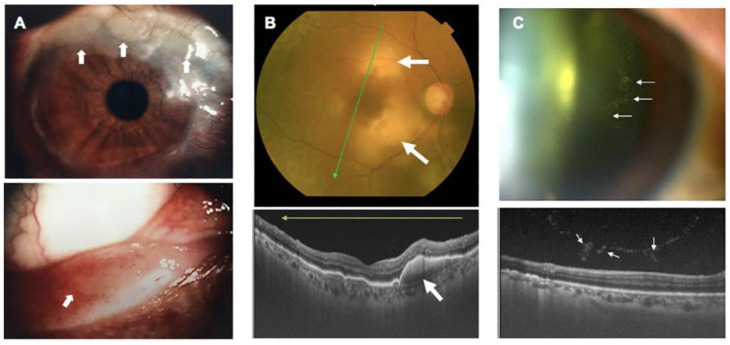
Ocular infiltration in ATL. Multiple knob-like ATL cell ocular infiltrates are seen (KAMOI sign). The KAMOI sign (**A**,**B**; white arrows) can be seen at the bulbar conjunctiva around the corneal limbus and at the palpebral conjunctiva around the lacrimal punctum (**A**) and in the retina (**B**). Multiple infiltrating ATL cells in the vitreous tend to form clusters (**C**) (Reproduced with permission from Kamoi et al., *Cornea* 2016 [62]; Kamoi. *Front. Microbiol*. 2020 [19]).

The most frequently seen manifestation is intraocular infiltration of ATL cells. We recently found that infiltration of ATL cells in the eye produces a specific sign, which we termed the “knob-like ATL cell multiple ocular infiltration” (KAMOI) sign, from the ocular surface to the retina [19,62,63] (Figure 3). This was identified using the recently developed imaging technology of ocular coherence tomography. The diagnosis of infiltration was facilitated by using a multiplex and broad-range polymerase chain reaction (PCR) system developed by us, which offers a precise differential diagnosis [64,65,66,67,68]. The therapy for ocular infiltration previously mainly relied on systemic ATL therapy, although the efficacy of topical treatment with the intra-vitreous injection of methotrexate in combination with focal radiation has been recently reported [19,63].

The second-most frequent ocular manifestation in ATL patients is CMV retinitis [61] (Table 3). CMV is well established as the most frequent pathogen underlying opportunistic infections in ATL patients [69,70], which also causes eye conditions. For diagnosis, multiplex and broad-range PCR of ocular samples are useful because ATL patients are typically immunocompromised, and other types of opportunistic infections (such as fungal infection) should also be excluded. In terms of other ATL-specific features, ocular inflammation developing simultaneously with ATL was previously reported [36,71], which correlated with an increase in ATL cells in the peripheral blood. This type of ocular inflammation is called ATL cell-induced uveitis [71].

**Figure 4:**
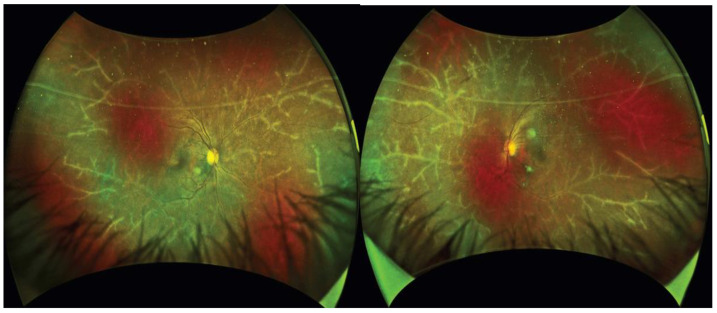
Frosted branch angiitis after allogenic hematopoietic stem-cell transplantation (Reproduced with permission from Kamoi et al., *Lancet Haematol.* 2020 [75]).

Recently, a new ocular finding was reported in the eye during the course of ATL. ATL is a life-threatening hematological disease with a poor prognosis that was previously reported to have a poor survival rate despite chemotherapy [72]. Recently, allogenic hematopoietic stem-cell transplantation (allo-HSCT) has been introduced for the treatment of ATL, which has resulted in an improvement in the overall survival of ATL patients [73]. However, a series of allo-HSCT procedures might disrupt immune homeostasis, resulting in systemic inflammation [74]. Our routine observation of the eyes of ATL patients before and after allo-HSCT with long-term follow up demonstrated that the activation of the immune system after allogeneic HSCT might cause intraocular inflammation, resulting in frosted branch angiitis, which might precede inflammation at other sites and organs [75] (Figure 4). This implies that appropriate monitoring of the intraocular condition should be considered after allogeneic HSCT for ATL.

## 8. Conclusions

Recently, several long-standing unresolved issues related to HU, such as its transmission route and factors associated with its development, have been clarified. Frequent and new ocular manifestations of HTLV-1-associated ATL have also been identified. In addition, a nationwide survey raised new issues about HU and addressed several concerns, such as the safety of biologics in terms of the eye in the presence of HTLV-1 infections. However, information on HU is still insufficient compared to that on ATL and HAM. Further clinical and basic investigations of HU are needed to prevent vision loss among HTLV-1-infected individuals.

## Figures and Tables

**Table 1:** Timing of testing for HTLV-1 antibodies.

Timing of Testing for HTLV-1 Antibodies	Northern (n = 4)	Central/Metropolitan (n = 40)			Southern (n = 16)		Total
	Hokkaido/Tohoku (n = 4)	Kanto (n = 22)	Chubu (n = 7)	Kinki (n = 11)	Chugoku/Shikoku (n = 7)	Kyushu (n = 9)	
Carried out routinely in considering differential diagnoses for uveitis	50.0%	52.5%			84.6%		58.3%
	50.0%	50.0%	57.1%	54.5%	75.0%	88.9%	
When ocular manifestation such as HAU, ATL, or HAM is suspected	50.0%	47.5%			25.0%		41.7%
	50.0%	50.0%	42.9%	45.5%	42.9%	11.1%	
When requested by Patient	0.0%	7.5%			0.0%		5.0%
	0.0%	4.5%	0.0%	18.2%	0.0%	0.0%	
When administering Steroid	25.0%	2.5%			0.0%		3.3%
	25.0%	0.0%	0.0%	9.1%	0.0%	0.0%	
When administering Immunosuppressant	25.0%	2.5%			0.0%		3.3%
	25.0%	0.0%	0.0%	9.1%	0.0%	0.0%	
When administering biological product	20.0%	0.0%			0.0%		1.7%
	20.0%	0.0%	0.0%	0.0%	0.0%	0.0%	
When performing Surgery	0.0%	5.0%			0.0%		3.3%
	0.0%	0.0%	14.3%	9.1%	0.0%	0.0%	
Others	0.0%	10.0%			0.0%		6.7%
	0.0%	18.2%	0.0%	0.0%	0.0%	0.0%	

Our nationwide survey identified that testing for HTLV-1 antibodies was routinely performed when considering the differential diagnosis of uveitis among 58.3% of facilities in Japan (Reproduced with permission from Kamoi et al., *Br. J. Ophthalmol*. 2020 [20]).

**Table 2:** Testing for HTLV-1 antibodies when an HTLV-1-related ocular manifestation is suspected.

Test for HTLV-1 Antibodies in Considering Differential Diagnoses for Uveitis	Northern (n = 5)	Central/Metropolitan (n = 47)			Southern (n = 16)		Total
	Hokkaido/Tohoku (n = 5)	Kanto (n = 26)	Chubu (n = 9)	Kinki (n = 12)	Chugoku/ Shikoku (n = 7)	Kyushu (n = 9)	
Yes	80.0%	82.9%			100.0%		86.8%
	80.0%	80.1%	77.8%	66.7%	100.0%	100.0%	
No/Un-identified	20.0%	17.1%			0.0%		13.2%
	20.0%	19.9%	22.2%	33.3%	0.0%	0.0%	

Our nationwide survey identified that testing for HTLV-1 antibodies was performed in 86.8% of facilities in Japan when an HTLV-1-related ocular manifestation was suspected (Reproduced with permission from Kamoi et al., *Br. J. Ophthalmol*. 2020 [20]).

**Table 3:** Nationwide survey of ATL-related ocular manifestations.

ATL-Related Ocular Manifestations	Number	%
Intraocular infiltration	22	45.8
Opportunistic infection	19	39.6
Cytomegalovirus	(19)	(100.0)
Herpesvirus	(2)	(10.5)
Toxoplasma	(1)	(5.3)
Dry eye	3	6.3
Scleritis	2	4.2
Uveitis	1	2.1
Anemic retinopathy	1	2.1
Total	48	100

Intraocular infiltration is the most frequent manifestation, followed by opportunistic infection (Reproduced with permission from Kamoi et al., *Front. Microbiol*. 2018 [61]).

## Data Availability

All data related to this study are presented and published here.
